# Spine deviations and orthodontic treatment of asymmetric malocclusions in children

**DOI:** 10.1186/1471-2474-13-151

**Published:** 2012-08-21

**Authors:** Carsten Lippold, Tatjana Moiseenko, Burkhard Drerup, Markus Schilgen, András Végh, Gholamreza Danesh

**Affiliations:** 1 Poliklinik für Kieferorthopädie, Universität Münster, Waldeyerstr. 30, Münster, 48149, Germany; 2 Klinik und Poliklinik für Technische Orthopädie und Rehabilitation, Universität Münster, Robert-Koch-Straße 30, Münster, 48149, Germany; 3 Akademie für Manuelle Medizin an der, Universität Münster, Waldeyerstr. 30, Münster, 48149, Germany; 4 Department of Orofacial Orthopedics and Orthodontics, Heim Pál Children’s Hospital, Ulloi ut 86, Budapest, 1089, Hungary; 5 Poliklinik für Kieferorthopädie, Universität Witten/Herdecke, Alfred-Herrhausen-Str. 45, Witten, 58455, Germany

## Abstract

**Background:**

The aim of this randomized clinical trial was to assess the effect of early orthodontic treatment for unilateral posterior cross bite in the late deciduous and early mixed dentition using orthopedic parameters.

**Methods:**

Early orthodontic treatment was performed by initial maxillary expansion and subsequent activator therapy (Münster treatment concept). The patient sample was initially comprised of 80 patients with unilateral posterior cross bite (mean age 7.3 years, SD 2.1 years). After randomization, 77 children attended the initial examination appointment (therapy = 37, control = 40); 31 children in the therapy group and 35 children in the control group were monitored at the follow-up examination (T2). The mean interval between T1 and T2 was 1.1 years (SD 0.2 years). Rasterstereography was used for back shape analysis at T1 and T2. Using the profile, the kyphotic and lordotic angle, the surface rotation, the lateral deviation, pelvic tilt and pelvic torsion, statistical differences at T1 and T2 between the therapy and control groups were calculated (*t*-test).

Our working hypothesis was, that early orthodontic treatment can induce negative therapeutic changes in body posture through thoracic and lumbar position changes in preadolescents with uniltaral cross bite.

**Results:**

No clinically relevant differences between the control and the therapy groups at T1 and T2 were found for the parameters of kyphotic and lordotic angle, the surface rotation, lateral deviation, pelvic tilt, and pelvic torsion.

**Conclusions:**

Our working hypothesis was tested to be not correct (within the limitations of this study). This randomized clinical trial demonstrates that in a juvenile population with unilateral posterior cross bite the selected early orthodontic treatment protocol does not affect negatively the postural parameters.

**Trial registration:**

DRKS00003497 on DRKS

## Background

The stomatognathic system is anatomically linked to the cervical vertebrae such that changes in the mouth, jaws and closely related structures can affect body posture [1-4]. Indeed, preadolescent patients requiring orthopedic treatment are known to have a higher high rate of malocclusions (83-87%) [1]. According to Solow et al. [3] the “soft tissue stretching hypothesis” emphasizes the functional influence of cervical posture on maxillo-mandibular growth. A correlation between increases in cervical lordosis and maxillofacial growth has been demonstrated [5]. This is supported by observations that suggest a correlation between the growth patterns of the mandible and the cranium, as well as between the vertical type of the mandible itself and the sagittal back contour parameters such as Fleche Cervicale, Fleche Lombaire and trunk inclination. Such observations suggest a possible interaction between craniofacial growth and spinal development. However, a better understanding of these mechanisms will require developmental studies that analyze the possible influence of orthodontic treatment on both the craniofacial complex and body posture. Mandibular asymmetries presenting with lateral cross bite are of particular interest due to the high incidence of orthopedic posture irregularities in affected patients [2]. The therapeutic impact of manipulating mandibular position has been assessed by Bracco et al. [6]. The effects of different jaw relations on body posture were studied in a sample of 95 subjects with a computerized footboard for posturometric and stabilometric analysis. Intriguingly, body posture was found to vary with jaw position. The results of this study confirmed that significant improvements in postural balance could be achieved with a myocentric position of the jaws.

In our own recent study [7], we established that therapeutic improvements in mandibular condyle position could be achieved using orthodontic treatment for functional unilateral posterior cross bite in a sample consisting of 65 children (6.9 ± 2.0 years of age) with late deciduous and early mixed dentition. Of the 65 children used in this randomized clinical trial, 31 underwent early orthodontic treatment and 34 did not. A three-dimensional ultrasound based assessment (Arcus Digma) of deviations between maximum intercuspidation and centric position was carried out at the beginning and at the end of treatment, and the condylar deviations between the groups were found to be significantly reduced in the treatment group. In contrast, the untreated subjects showed no spontaneous self-healing tendencies. The interaction between orthodontic treatment of unilateral cross bite and mandibular condyle position is of clinical interest given the positive influence that such an intervention can have on further preadolescent craniofacial growth.

The aim of the present trial was to assess the therapeutic effect of an early orthodontic treatment protocol in patients with unilateral posterior cross bite [8-10] on body posture, as measured through several parameters. We hypothesized that early orthodontic treatment can induce negative therapeutic changes in body posture through thoracic and lumbar position changes in preadolescents with uniltaral cross bite. To assess body posture, we utilized a study protocol incorporating optical 3-D back shape measurement.

## Methods

### Subjects

**Figure 1:**
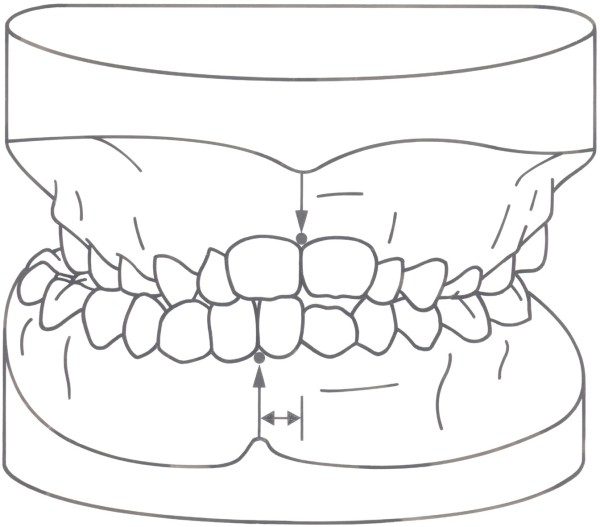
**Lateral Crossbite.** Asymmetric dental occlusion: posterior crossbite in the early mixed dentition with resulting midline deviation due to the inherent functional asymmetries of the mandibular system.

The investigation was planned as a randomized clinical trial and was approved by the local ethics committee with reference number “2IXEhm” (Ethics Committee of the Medical Faculty, Westfälische-Wilhelms-Universität Münster, Germany) and registred with the number: DRKS00003497. The study protocol was established and the number of patients needed for adequate statistical power was determined through assistance from Zentrum für Klinische Studien Münster (ZKS). A minimum of 30 patients each for the therapy and control group was established. Patients to be included were required to have late deciduous and early mixed dentition, unilateral posterior cross bite and functional mandibular asymmetry (according to Figure 1). Patients with previous orthodontic treatment, ongoing habits, systemic illness under long-term therapy (e.g., diabetes mellitus), syndromes, cleft lip and palate, physical or mental handicaps and known structural orthopedic illnesses (e.g., Scheuermann’s disease, stiff neck) and spinal deformities were excluded. The subjects were screened for sport habits before and during the orthodontic therapy and it was recommended to them, not to change the initial sports habits in order to minimize this functional factor in the study. Parents gave their informed consent according to the requirements of the local ethics committee and the Helsinki criteria. The patients were initially randomized using block randomization (block length 20; allocation ratio 1:1) to either the control or therapy group. A total of 82 children (38 boys and 44 girls) met the above criteria; 40 were assigned to the therapy group, 42 were assigned to the control group and 77 children attended the initial examination appointment. Due to various personal reasons, 5 children dropped out after randomization. A total of 37 children remained in the therapy group, and 40 children remained in the control group. For the final examination, 11 children were excluded from the study for either personal reasons or for being unable to keep to the mandatory time schedule. Thus 66 children (30 boys and 36 girls) remained: 31 in the therapy group (13 boys and 18 girls) and 35 in the control group (17 boys and 18 girls). The children’s mean age was 7.3 (SD 2.1 years) at the beginning of the study and 8.3 years (SD 2.1) at the end of the study. The gender ratio was nearly equal in the groups. For all patients, two examination appointments were fixed: an initial examination appointment (T1) and a final examination appointment one year later (T2).

### Orthodontic treatment

**Figure 2:**
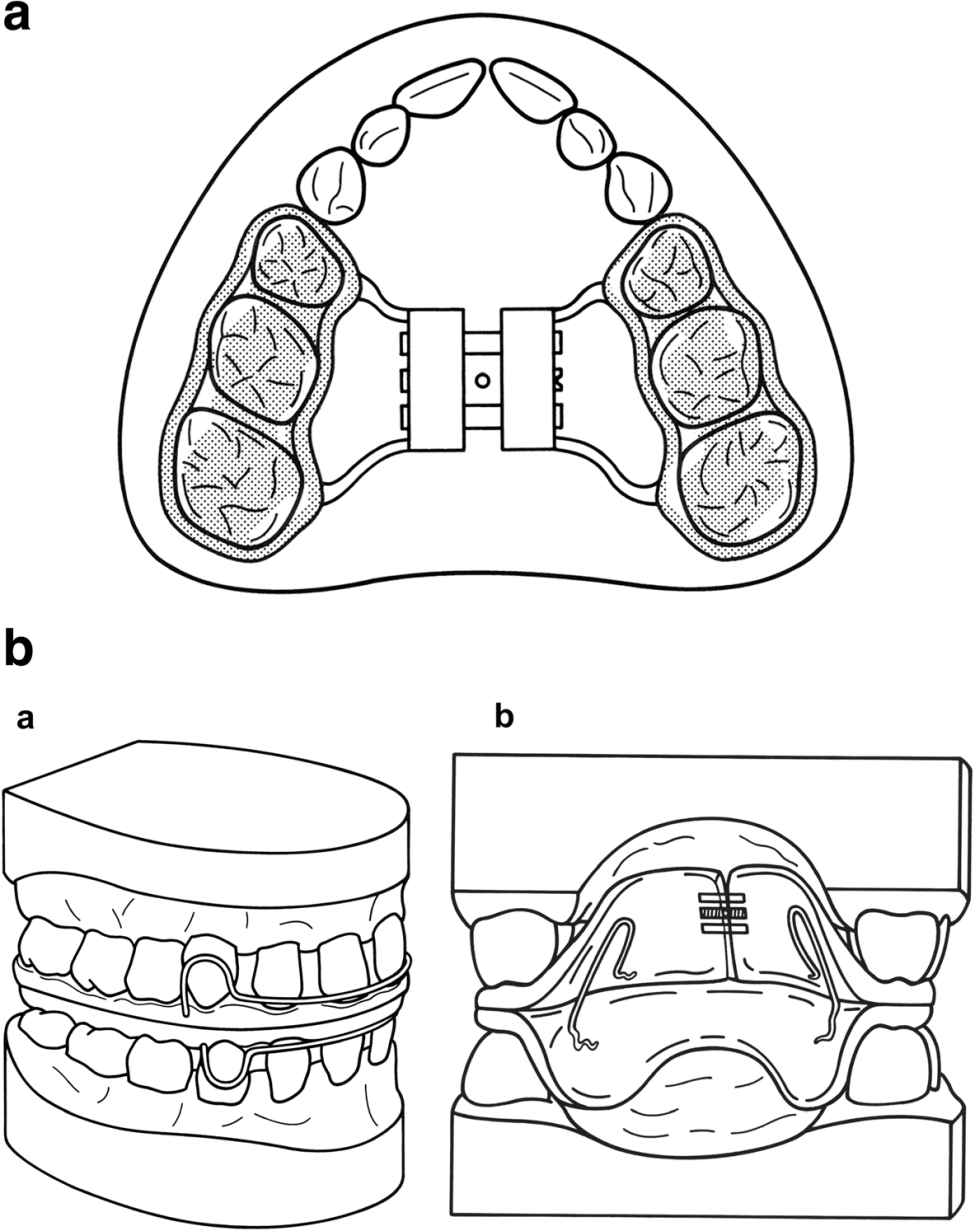
**Orthodontic Appliance for Maxillary Expansion.** Palatal expansion appliance used for slow expansion of the maxillary bones, bonded on the posterior teeth. **b** Orthodontic Appliance for Functional Rehabilitation. U-Bow activator Type 1, as described by Karwetzky, used to achieve midline coordination and retain palatal expansion (a - outer view, b – inner view).

In the therapy group for slow expansion of the maxillary bone formation, a bonded palatal expansion appliance (Figure 2a) was used as previously described by McNamara et al. [11]. After correction of the maxillary discrepancy, an orthodontic activator treatment (U-Bow activator Type 1) as described by Karwetzky [12] was applied to achieve midline coordination and to retain the amount of palatal expansion (Figure 2b). Details are given by Lippold et al. [7].

### 3-D back shape measurement

To measure back shape and to determine three-dimensional orthopedic parameters of the back and spine, rasterstereography (Formetric 2, Diers International GmbH, Schlangenbad, Germany) was used. This optical contact-free photogrammetric method provides high accuracy of the surface data and good correlation with radiological findings [5,13-15] for the spine reconstruction, but without the risk of radiation hazards. The recording required only 0.04 sec with the subject standing free without pads or trunk fixation.

Evaluating the record was accomplished in three steps. First, the back shape was reconstructed by photogrammetric methods that generated a list of 3-D coordinate data of back surface points in a regular array. Second, three anatomical landmarks – the vertebra prominens and the two spina iliaca posterior superior (lumbar dimples) – were detected and localized using automated mathematical procedures that scanned the reconstructed surface for its characteristic shape. When these three landmarks were localized, they spanned a body fixed coordinate system, which provided an objective and automated determination of the longitudinal, sagittal and lateral direction. Third, the symmetry line of the back was determined by mathematical shape analysis and model calculation based on the 3-D reconstruction of the back surface. The symmetry line is a reasonable estimate of the line of spinous processes [16]. Finally, the lateral projection of the symmetry line, which is virtually the sagittal back profile, was calculated and analyzed such that the appropriate shape parameters for the back could be determined.

The automated mathematical evaluation procedures necessitate a high level of accuracy of the input data: back shape and the sagittal profiles were therefore recorded with 0.25 mm accuracy. The precision in localizing the vertebra prominens landmark and the dimple landmarks was of high accuracy according to Hierholzer [17].

**Figure 3:**
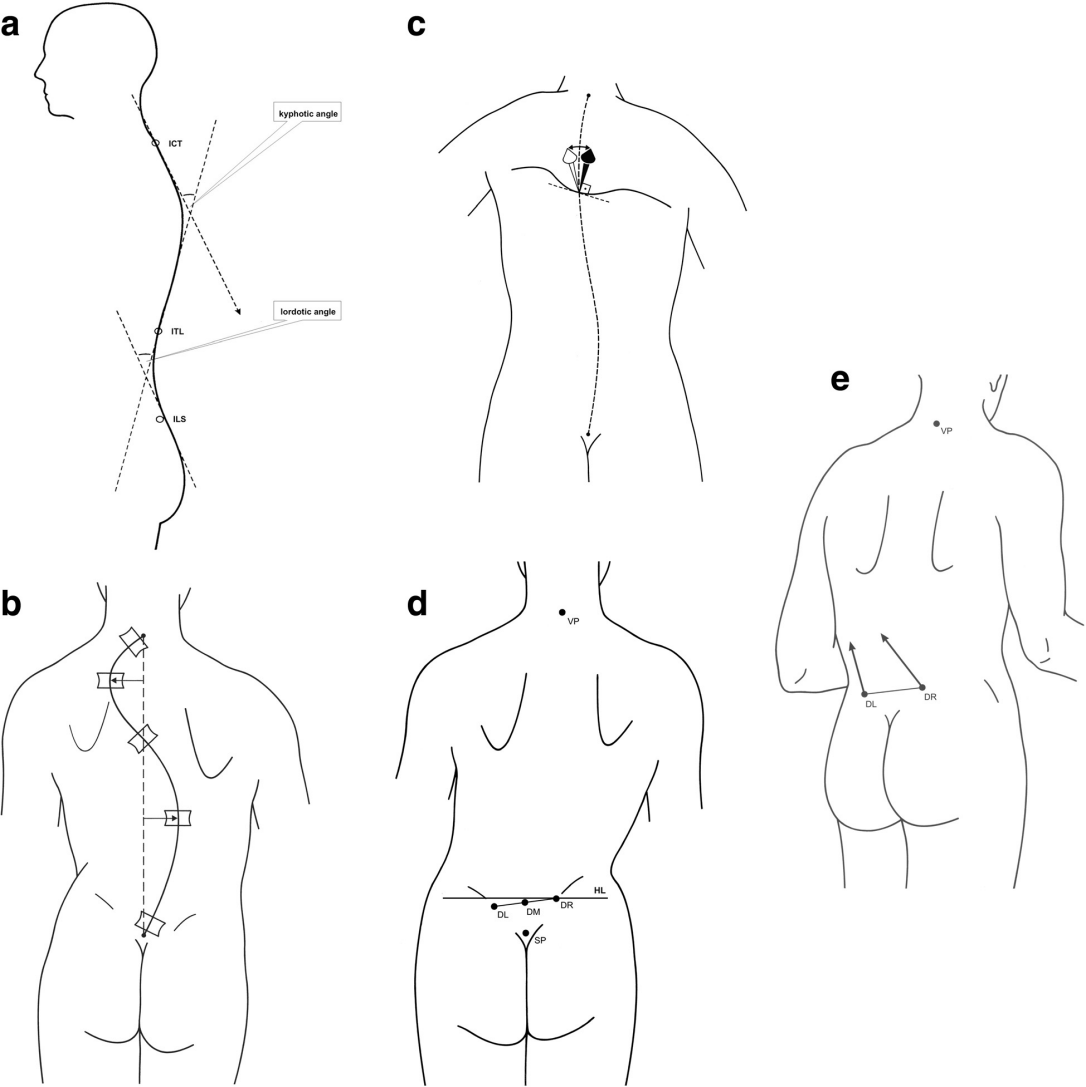
**Rasterstereographic Analysis: Kyphotic and Lordotic Angle.** Kyphotic and lordotic angles were calculated from geometric analysis of the sagittal profile, which provides the points of inflection and their respective inflection tangents in the cervico-thoracic transition (ICT), the thoracic-lumbar transition (ITL) and the lumbar-sacral transition (ILS). The kyphotic and lordotic angles are each spanned by two of the inflectional tangents. **b** Rasterstereographic Analysis: Lateral Deviation. Lateral deviation refers to the distance between the center of the reconstructed vertebral body and the sagittal plane at a given vertebral level. **c** Rasterstereographic Analysis: Vertebral Rotation. Vertebral rotation at a given level was estimated from surface rotation at the pertinent point of the symmetry line, using the sagittal direction as a reference. **d** Rasterstereographic Analysis: Pelvic Tilt. Pelvic tilt was calculated from the height difference of the two lumbar dimples. **e** Rasterstereographic Analysis: Pelvic Torsion. Pelvic torsion was calculated from the difference of surface orientations in the lumbar dimples. It has a positive value with posterior rotation of the right pelvic side and an anterior rotation of the left side.

Shape parameters of the back were calculated from the sagittal profile and from the back shape. Geometric analysis of the sagittal profile was used to determine the kyphotic and lordotic angle. Geometric analysis of the profile provided the points of inflection and their respective inflection tangents in the cervico-thoracic transition (ICT), thoracic-lumbar transition (ITL) and lumbar-sacral transition (ILS). The kyphotic and lordotic angles are spanned by two of the inflectional tangents each (Figure 3a). Additional parameters used in the characterization of back and spinal shape were the lateral deviation, the vertebral rotation, the pelvic tilt and the pelvic torsion [16]. These parameters rely on biomechanical modeling of the spine and on the shape analysis of the back with methods from differential geometry [18] and are consistent with radiological findings [14].

Lateral deviation suggests that at a given vertebral level, the distance between the center of the reconstructed vertebral body and the sagittal plane (Figure 3b). Here, the parameter specifies the mean values of the measured distances between the vertebra prominens and the lumbar dimples midline.

Vertebral rotation at a given level was estimated from surface rotation at the pertinent point of the symmetry line, with the sagittal direction as the reference direction (Figure 3c). Again, this parameter specifies the mean value over the same distance. Pelvic tilt was calculated from the height difference of the two lumbar dimples (Figure 3d); similarly, pelvic torsion was calculated from the difference of surface orientations in the lumbar dimples (Figure 3e). The latter has a positive value with posterior rotation of the right side of the pelvis and an anterior rotation of the left side of the pelvis. In the reversed configuration, the sign of the pelvic torsion is negative.

### Data analysis

Statistical processing was performed with SPSS 12.0 (Lead Tech., Chicago, USA) under biomathematical assistance by “Zentrum für Klinische Studien Münster (ZKS)” at our university. The therapy and control groups were tested for normal distribution using the Kolmogorow-Smirnow test. SPSS 12.0 (Lead Tech., Chicago, IL, USA) software was used in the data analysis. The posture parameters involved in the measurements were as follows: UTI, KA, LA and PI. To determine the craniofacial morphology, the Angle Classification and the overjet were considered. ANOVA, Scheffé and Kruskal-Wallis procedures were used to test our hypothesis. Significance was set at p < 0.05.

## Results

The test for normal distribution between the therapy and the control group revealed no significant differences (with the significance set at *p* < 0.05); thus the paired *t*-test could be used to assess significant differences between the two groups. At the beginning of the study (T1) no significant differences were detected regarding the shape parameters of the back (Table 1).

**Table 1:** Differences between the control and the therapy group in terms of the shape parameters of the back as measured at T1 and T2

	**kyphothic angle [°]**			**surface rotation [°]**			**pelvic tilt [°]**		
	**Mean (SD)**			**Mean (SD)**			**Mean (SD)**		
	**Control**	**Therapy**	***P*- value**	**Control**	**Therapy**	***P*- value**	**Control**	**Therapy**	***P*- value**
**T 1**	39,5 (7,1)	39,1 (6,1)	n. s.	−0,3 (4,5)	−1,5 (4,5)	n. s.	−0,9 (2,2)	0,1 (2,9)	n. s.
**T 2**	40,1 (7,3)	38,1 (6,8)	n. s.	0,5 (3,9)	0,2 (4,5)	n. s.	−0,5 (2,0)	−0,8 (2,5)	n. s.

*Significant n.s. = not significant.

The results of the statistical analysis are presented in Tables 1 and 2.

**Table 2:** Differences of the shape parameters of the back between the control and the therapy groups, measured after completing the orthodontic treatment (T1 – T2)

	**lordotic angle [°]**			**lateral deviation [mm]**			**pelvic torsion [°]**		
	**Mean (SD)**			**Mean (SD)**			**Mean (SD)**		
	**Control**	**Therapy**	***P*- value**	**Control**	**Therapy**	***P*- value**	**Control**	**Therapy**	***P*- value**
**T 1**	33,5 (8,1)	33,7 (7,3)	n. s.	3,1 (1,9)	2,6 (1,4)	n. s.	0,4 (2,0)	0,3 (1,9)	n. s.
**T 2**	34,9 (8,1)	34,3 (6,6)	n. s.	2,9 (1,5)	2,0 (1,7)	n. s.	0,5 (1,9)	0,8 (1,8)	n. s.

*Significant n.s. = not significant.

The kyphotic angle in the control and therapy group was nearly the same at the beginning of the study. In the control group a slight increase of kyphotic angle was measured. In the therapy group, a moderate reduction was observed. While there was no significant difference between T1 and T2 in the two groups, the difference between the two groups increased significantly between T1 and T2 (*p* = 0.047). However, no significant differences were measured at T2 between the control and the therapy group.

The surface rotation did not change significantly in the control group between T1 and T2. A statistically significant reduction (*p* = 0.029) between T1 and T2 was observed for the therapy group, but this is clinically not relevant. There were no significant differences between the control and the therapy group at T2.

No significant differences were detected for the lordotic angle between T1 and T2 or between the control and the therapy group at T2. For lateral deviation the control group showed no significant differences between T1 and T2. In contrast, a statistically significant reduction (*p* = 0.030) was observed in the therapy group. The difference between the control and therapy group at T2 was not statistically different.

A moderate pelvic tilt was present in both the control and the therapy group at the beginning of the study (T1), which did not change significantly between T1 and T2 for either the control or the therapy group. However, the difference between the groups changed significantly (*p* = 0.040) and reversed its sign. At T2 no significant differences could be measured between the control and the therapy group.

The values for pelvic torsion were small and did not vary significantly between T1 and T2 for the control and therapy group. Between the control and the therapy group no statistically significant differences were detected at T2.

## Discussion

The relationship between unilateral posterior cross bite, asymmetries in the craniofacial complex and postural disorders has been previously reviewed by Korbmacher et al. [1]. As noted above, the current literature is lacking in randomized clinical trials addressing the development of the dental and craniofacial complex and its possible influence on the morphology of the cranio-cervical and vertebral column. Although the close functional and morphological relationship between the stomatognathic system and the vertebral column have been described [2-4,6] to date no randomized trials have addressed the possible impact of that treatment of unilateral posterior cross bite could have on the vertebral column.

Our working hypothesis for this study was that early orthodontic treatment in preadolescents with unilateral cross bite could induce a negative change in vertebral alignment with respect to the thoracic and lumbar spine sections. To test this hypothesis, we analyzed the parameters quantifying the sagittal curvature in the kyphotic and lumbar spine in a randomized clinical trial, using rasterstereography to evaluate possible effects of treatment. Additionally, we assessed frontal plane effects based on surface rotation and lateral deviation. In this study, we found that in both the therapy and the control group, the kyphotic and the lordotic angle were within the limits of clinical tolerance. A significant difference was observed in the kyphotic angle between initial (T1) and follow-up (T2) examination; while in the control group the kyphotic angle slightly increases, the converse is observed in the therapy group. However, a change of 1 to 2 degrees in the kyphotic angle is not of clinical relevance, especially in a juvenile population.

No significant difference in the control and therapy group was observed in surface rotation. The control group and therapy group showed measurement values that did not differ significantly from the expected biomechanical value of zero. Nevertheless, in the therapy group, the distance from zero decreases between T1 and T2 – a decrease that is significant as assessed by the paired *t*-test. The distance from zero does not change significantly in the control group.

No significant changes were detected for the pelvic tilt, and no pelvic torsion was observed for the patient sample due to the small amount of pelvic tilt. The orthopedic data collected in this study reveal no manifest orthopedic illnesses; furthermore, no clinically relevant changes in the parameters of kyphotic angle, surface rotation and lateral deviation were observed.

Our results suggest that modern orthodontic treatments for unilateral posterior cross bite [19,20] does not influence negatively the thoracic spine. The orthodontic treatment had no impact on impairing postural parameters. Therapy had no negative effect on several parameters of spinal positioning, including kyphotic and lordotic angle, surface rotation, pelvic angles and lateral deviation. We therefore conclude that our working hypothesis – namely, that early orthodontic treatment can induce negative therapeutic changes in body posture through thoracic and lumbar position changes in preadolescents with uniltaral cross bite– is not valid within the limitations of this study.

While it has been hypothesized that orthodontic treatment can influence the cervical spine, it seems likely that in lower segments, these effects disappear due to biomechanical effects or by inherent measurement limitations. According to the theory of scoliosis progression postulated by White et al. [21], an imbalance in the thoracic spine can result in imbalanced loading of the spinal muscles and ligaments that finally results in a progression to scoliosis. Solow et al. proposed a similar theory about the development of the cranio-cervical complex based on the individual muscular balances [3]. Local imbalances are suggested to contribute to the development of idiopathic scoliosis in the preadolescent growth phase [21,22].

The effects of asymmetric loads on the spinal system were suggested by Roaf to be the major contributing factor in the development of juvenile idiopathic scoliosis [23]. Idiopathic scoliosis in the preadolescent growth phase is believed to be reversible based on work in animal models [24]. We therefore conclude that early treatment of unilateral cross bite is advisable since it does affect negatively the vertebral column. After the adolescent growth spurt, conservative correction of spinal postural disorders seems to be more difficult [25,26].

## Conclusion

We conclude that our working hypothesis – namely, that early orthodontic treatment can induce negative therapeutic changes in body posture through thoracic and lumbar position changes in preadolescents with uniltaral cross bite– is not valid within the limitations of this study.

This randomized clinical trial demonstrates that in a juvenile population with unilateral posterior cross bite the selected early orthodontic treatment protocol does not affect negatively the postural parameters.

Additional studies will be necessary to further analyze the clinical biomechanical background.

## Competing interests

The authors have no competing interests to report.

## Authors’ contributions

CL and GD designed and initiated the study. TM collected the data on the orthodontic 3-D models and assisted in drafting the manuscript. GD performed orthodontic analysis, data collection, data processing and statistical analysis. MS participated in the study design, performed the data collection for the rasterstereographic analysis. BD participated study design, and programmed the raterstereographic units, BD also transferred data into the statistical program and helped draft the manuscript. AV provided the idea for digital plastermodel analysis, performed some of the data analysis and helped to draft the manuscript. All authors read and approved the final manuscript.

## Pre-publication history

The pre-publication history for this paper can be accessed here:

http://www.biomedcentral.com/1471-2474/13/151/prepub
